# Silica from diatom frustules as anode material for Li-ion batteries†

**DOI:** 10.1039/c9ra07271c

**Published:** 2019-12-12

**Authors:** Andreas Nicolai Norberg, Nils Peter Wagner, Henning Kaland, Fride Vullum-Bruer, Ann Mari Svensson

**Affiliations:** Department of Materials Science and Engineering, Norwegian University of Science and Technology, NTNU Sem Sælands Vei 12, Realfagbygget K2-132 7034 Trondheim Norway andreas.n.norberg@ntnu.no henning.kaland@ntnu.no annmari.svensson@ntnu.no; Department of Sustainable Energy Technology, SINTEF Industry Strindveien 4 7034 Trondheim Norway nils.peter.wagner@sintef.no; Department of Thermal Energy, SINTEF Energy Sem Sælands Vei 11 7034 Trondheim Norway fride.vullum.bruer@ntnu.no

## Abstract

In spite of its insulating nature, SiO_2_ may be utilized as active anode material for Li-ion batteries. Synthetic SiO_2_ will typically require sophisticated synthesis and/or activation procedures in order to obtain a satisfactory performance. Here, we report on diatom frustules as active anode material without the need for extensive activation procedures. These are composed primarily of silica, exhibiting sophisticated porous structures. Various means of optimizing the performance were investigated. These included carbon coating, the addition of fluoroethylene carbonate (FEC) and vinylene carbonate (VC) to the carbonate-based electrolyte, as well as activation by an initial potentiostatic hold step. The highest capacity (723 mA h g^−1^) was obtained with composite electrodes with pristine diatom frustules and conventional carbon black as additive, with the capacity still increasing after 50 cycles. The capacity was around 624 mA h g^−1^ after subtraction of the contributions from the carbon black. Carbon coated diatom frustules showed a slightly lower but stable capacity after 50 cycles (600 mA h g^−1^ after subtraction of contributions from the carbon coating and the carbon black). By the use of electrochemical characterization methods, as well as *post-mortem* studies, differences in reaction mechanisms could be identified and attributed to the operating and processing parameters.

## Abstract 2

Silica derived from algae was used as anode material in Li-ion batteries, giving a capacity of more than 700 mA h g^−1^.

## Graphical abstract

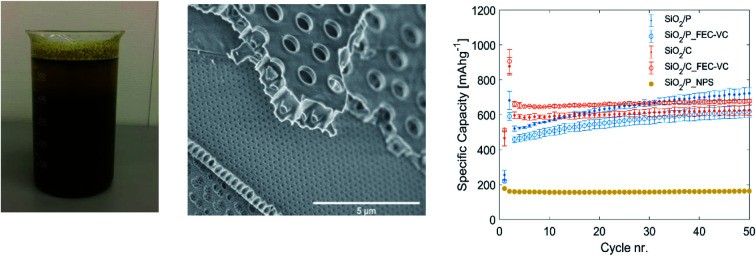

## 1. Introduction

Rechargeable lithium-ion (Li-ion) batteries are already widely used and are the dominating solution for automotive as well as portable consumer devises and high-power applications due to the superior power and energy density. However, several challenges of Li-ion battery technology are yet to be overcome, *e.g.* with respect to cost, resource availability, and safety issues related to risk of thermal runaway of the batteries.^1–3^ Another issue with Li-ion batteries is the relatively poor environmental footprint related to the production of the batteries.^4^ Since Li-ion batteries are expected to dominate the battery market for portable electronics as well as electric vehicles in the foreseeable future, it is important to address and solve the above-mentioned issues by for example developing and identifying new electrode materials as well as other components used in the production of a complete battery. Current state-of-the-art anode materials (graphites) have a specific theoretical capacity of 372 mA h g^−1^ and are used in most conventional Li-ion batteries.^5^ Approximately 10% of this capacity is irreversibly lost in the first cycle due to decomposition of electrolyte on the graphite surface. Recently, small amounts (∼5–12 wt%) of silicon (Si) or SiO_*x*_ is mixed into the graphite anode for a small increase in battery capacity.^6–8^ Significant research effort during the past 10 years has been focused on developing Si-graphite composites and Si as the active material, due to its extremely high volumetric and gravimetric capacities, which is up to 10 times that of graphite.^9^ However, due to the issues caused by large volume expansion during cycling and excessive SEI formation, the implementation of Si anodes is yet to be realized in commercial batteries. It is foreseen, that the next generation of Li-ion batteries, the so-called generation 3b, will have Si-graphite composite anodes with a higher content of Si than state-of-the art Li-ion batteries.^10^ From an environmental perspective, there are also challenges associated with Si that needs to be addressed. Most of the pure grade Si in the world is obtained from crystalline silica (SiO_2_) through a reaction with carbon at elevated temperatures, resulting in a high CO_2_ footprint in the range 3.5 to 11 kg CO_2_ per kg Si produced (depending on origin of the energy used). Battery grade graphite is either natural graphite (39% of the marked), or synthetic graphite, produced from residues from oil refineries. Natural graphite is on the list of critical raw materials for battery production,^11^ while synthetic graphite is also associated with significant CO_2_ emissions, as it is made from the residues from oil refineries.

As an alternative to Si, SiO_2_ has emerged as a potential abundant and environmentally benign anode material with an estimated capacity between 749 and 1673 mA h g^−1^.^12^ Initially, SiO_2_ was believed to be electrochemically inactive towards Li. However, in 2001 Gao *et al.* reported that SiO_2_ nanoparticles were reactive towards Li-ions at potentials between 0 V and 1 V.^13^ Sun *et al.* was the first to propose a lithiation mechanism, which was further expanded on by Guo and Chang *et al.* resulting in the following proposed mechanisms:^12,14,15^

1 5SiO_2_ + 4Li^+^ + 4e^−^ ↔ 2Li_2_Si_2_O_5_ + Si

2 2SiO_2_ + 4Li^+^ + 4e^−^ → Li_4_SiO_4_ + Si

3 SiO_2_ + 4Li^+^ + 4e^−^ → 2Li_2_O + Si

4 Si + *x*Li^+^ + *x*e^−^ → Li_*x*_Si

By far the most common method for preparing SiO_2_ for battery anodes in the literature is the preparation of porous SiO_2_ structures by wet chemical methods.^16–24^ However, Chang *et al.* have also demonstrated that it is possible to achieve a capacity of approximately 750 mA h g^−1^, simply by milling quartz.^12^ In practice, nanostructures of silica have shown stable cycling capacity of up to 1000 mA h g^−1^ without suffering from similar performance issues as Si.^17^ The improved cycling stability of SiO_2_ compared to Si, is believed to originate from the formation of lithium oxide (Li_2_O) and lithium silicates, which buffer the expansion of Si during lithiation.^14,25,26^ To further limit the volume expansion of Si and improve the otherwise sluggish electronic conductivity of SiO_2_, different carbon coatings, or attempts to embed the SiO_2_ particles in a carbon matrix, are often utilized.^16,21,24,27–30^

In the work of Lepoivre *et al.*,^31^ two batches of pure and monodisperse SiO_2_ spherical particles with a diameter of 150/200 nm and 500 nm were prepared. A capacity of 400 mA h g^−1^ was obtained for electrodes made from the material with 200 nm particles, after a so-called potentiostatic discharge (potential held at 2 mV for 250 h prior to cycling). Thus, partial conversion of the material to silicon and Li_15_Si_4_ and Li_4_SiO_4_ was achieved. Full conversion was not observed, which was attributed to the insulating character of SiO_2_, preventing the reduction beyond a certain penetration depth (∼45/50 nm).^32,33^

The drawback with all these materials is that they require quite complex and expensive synthesis methods. On the other hand, diatom frustules found in the seabed are composed primarily of silica, exhibiting porous and sophisticated structures ranging in size from 10 nm to 1 μm.^34,35^ These structures might be beneficial to Li-ion batteries by easing electrolyte penetration and shortening the diffusion path for Li-ions between the electrolyte and the active material.^36,37^ One research group has previously reported the use of diatom frustules as active material in Li-ion batteries.^38–40^ Here, the diatom frustules were used in combination with red algae as anode material. Half-cells were tested with a capacity of 500 mA h g^−1^ after 80 cycles.

In this work, we investigate the use of a different kind of diatom frustules as anode material in Li-ion batteries and investigate various means of optimizing the performance. These include carbon coating, the addition of fluoroethylene carbonate (FEC) and vinylene carbonate (VC) to the carbonate-based electrolyte, as well as activation by an initial potentiostatic hold step. The highest capacity (723 mA h g^−1^) was achieved with composite electrodes with pristine diatom frustules, no electrolyte additives and conventional carbon black conductive additive. Using electrochemical methods, as well as *post-mortem* studies, differences in reaction mechanisms could be identified and attributed to the operating and processing parameters.

## 2. Experimental

### 2.1 Pre-processing of diatom frustules and materials preparation

The pre-processing and carbon coating of diatoms described in this work are based on the process described in a patent by Vullum-Bruer *et al.*^41^ Sea-hauled coscinodiscus diatoms (Planktonic AS) were separated from seawater by a 36 μm mesh sieve and dried for 24 h at 90 °C. The dried diatoms were rinsed under running deionized-water (DI water), heated on a hotplate to 130 °C and stirred at 500 rpm for two hours at a diatom to DI–water ratio of 1 : 100 wt%. Following heating, fresh DI–water was added at the same ratio and the beaker was sonicated for 30 min before the diatoms were washed under running DI–water for 5 min. Finally, the diatoms were dried at 90 °C for 24 h followed by 150 °C for 24 h and calcined in a tube furnace at 650 °C for 2 h in a synthetic air atmosphere. Calcined diatoms were milled by planetary milling at 600 rpm for 40 min at 10 min intervals with a 5 min break between the intervals. To investigate the effect of carbon coating, milled diatoms were mixed with sucrose (Sigma Aldrich) dissolved in DI–water as a carbon precursor with a ratio of 20 to 80 wt% silica and sucrose, respectively. The solvent was evaporated on a hotplate at 40 °C while stirring at 300 rpm, followed by a heat-treatment in a tube furnace at 650 °C under flowing Ar atmosphere. The pristine and carbon coated frustules are named SiO_2_/P and SiO_2_/C, respectively.

### 2.2 Materials characterization

Surface area and porosity data were determined by nitrogen adsorption (Micrometrics Tristar 3000). Samples were degassed for 12 h under vacuum at 250 °C prior to analysis. Particle size and particle size distribution were measured in solution by laser diffraction (Horiba Partica LA-960). To limit agglomeration, the sample solution was sonicated for 30 min prior to the measurement. The carbon content was measured by thermogravimetric analysis (TGA, Netzch STA 449C Thermal Analysis System) with a heating rate of 10 °C min^−1^.

Scanning electron microscopy (SEM, Hitachi S-5500) was used to study the morphology of the diatom frustules. Focused ion beam (FIB, FEI Helios Nanolab Dual Beam) cross-sections were prepared and electrodes analyzed by SEM (FEI Apreo) before and after cycling.

### 2.3 Electrode preparation

A slurry was prepared by mixing the active material, pristine SiO_2_ (SiO_2_/P) or carbon coated SiO_2_ (SiO_2_/C) with 35 wt% carbon black (CB, Timcal C-Nergy C65) and 15 wt% sodium alginate (Sigma Aldrich Sodium alginate). In order to investigate the capacity contribution of carbon, two reference electrodes were prepared with pyrolyzed sucrose and CB as the active material, otherwise the electrodes were the same composition. The slurries were mixed by ball milling for 45 min in stainless steel containers using a RETSCH mixer mill, casted on a 10 μm thick copper foil and dried overnight at 90 °C in a vacuum oven. Capacity is reported with respect to the mass of the SiO_2_ or the carbon coated SiO_2_, unless otherwise stated.

### 2.4 Electrochemical characterization

Electrochemical characterization was carried out in CR2016 coin cells. Li-foil was used as counter electrode and a Celgard 2400 as separator. Two electrolytes were used; a commercial 1 M LiPF_6_ in 50 : 50 vol% EC : DEC (Sigma Aldrich) and an in-house made electrolyte prepared by mixing the commercial electrolyte with fluoroethylene carbonate (FEC, Sigma Aldrich) and vinylene carbonate (VC, Sigma Aldrich) with the ratio 94 : 5 : 1 in vol%, respectively. Cells with the additives are named SiO_2_/P_FEC-VC and SiO_2_/C_FEC-VC. Galvanostatic cycling was performed using BCS 805 (Biologic) and CT2001A (Lanhe) galvanostats between 0.01 and 2.0 V *vs.* Li/Li^+^. In addition, an electrochemical reduction program was used by introducing a 48 h potentiostatic step at 2 mV *vs.* Li/Li^+^ in the second discharge cycle, in accordance with the procedure suggested by Lepoivre *et al.*^31^ This potential was chosen because it is below the lithiation potential of SiO_2_, but above the potential of Li-plating. The current density was 50 mA g^−1^ for the first cycle and the electrochemical reduction step, and 200 mA g^−1^ for the subsequent cycles. All cells had an active material mass of 0.40 mg, apart from the reference cell not subjected to a potentiostatic step, which had an active material mass of 0.45 mg.

## 3. Results and discussion

**Fig. 1:**
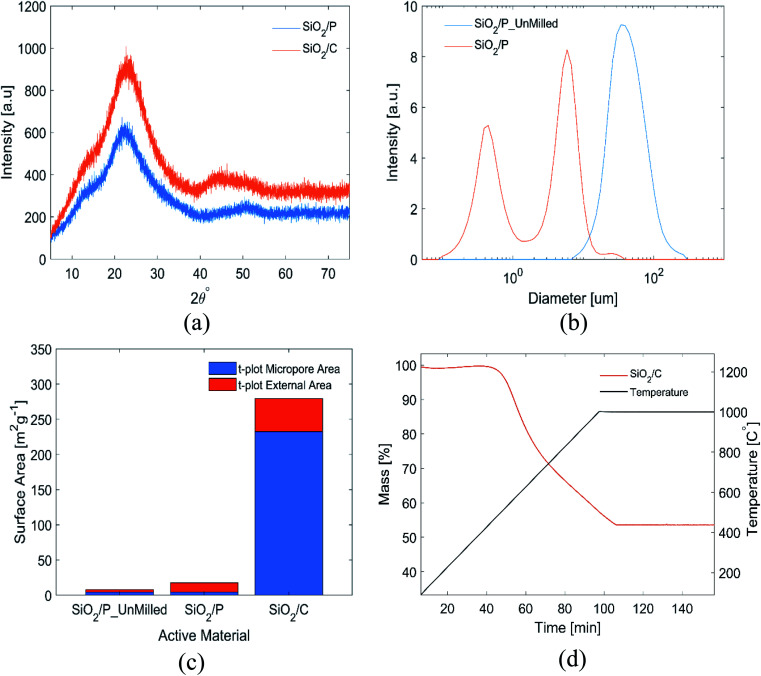
(a) XRD patterns of SiO_2_/P and SiO_2_/C extracted from diatom frustules, (b) size distribution of milled and un-milled SiO_2_/P obtained by laser diffraction, (c) surface area of SiO_2_/P_unMilled, SiO_2_/P and SiO_2_/C, (d) thermogravimetric curves of SiO_2_/C.

Results from the materials characterization of the pre-processed, as well as pre-processed and carbon-coated frustules are shown in Fig. 1. The XRD patterns in Fig. 1(a) show the absence of crystalline phases in the samples. The broad peak centered at 22° is associated with amorphous SiO_2_.^21,42^ EDX analysis of the frustules confirmed that the frustules mainly comprise of Si and O (Fig. S1†). However, small amounts of Na and Fe was identified. The presence of Na can be explained by the Na-alginate binder, while diatom frustules are known to comprise of small amounts of Fe.^43^ However, it is also possible that the Fe was introduced from the stainless steel jar used for slurry mixing. The particle size distributions of the milled and un-milled SiO_2_ determined by laser diffraction can be seen in Fig. 1(b). The mean and the median size of the milled material is 3.1 ± 0.2 and 3.8 ± 0.2 μm, respectively. As evident from Fig. 1(b), the milling results in a bimodal size distribution, with the maximum peak intensity of the two size distributions at 0.445 μm and 5.87 μm. The BET surface area and the *t*-plot areas, obtained from nitrogen adsorption data, are shown in Fig. 1(c). Milling of the SiO_2_ increased the BET surface area from 8.2 to 17.2 m^2^ g^−1^, and this increase was almost exclusively in external surface area. The carbon coated SiO_2_ shows a significant increase in BET surface area, to 289 m^2^ g^−1^. The increase in surface area is a result from an increase in both micropores and external area. The carbon content of the coated SiO_2_ was determined by TGA to be 46.5 wt% (Fig. 1(d)).

**Fig. 2:**
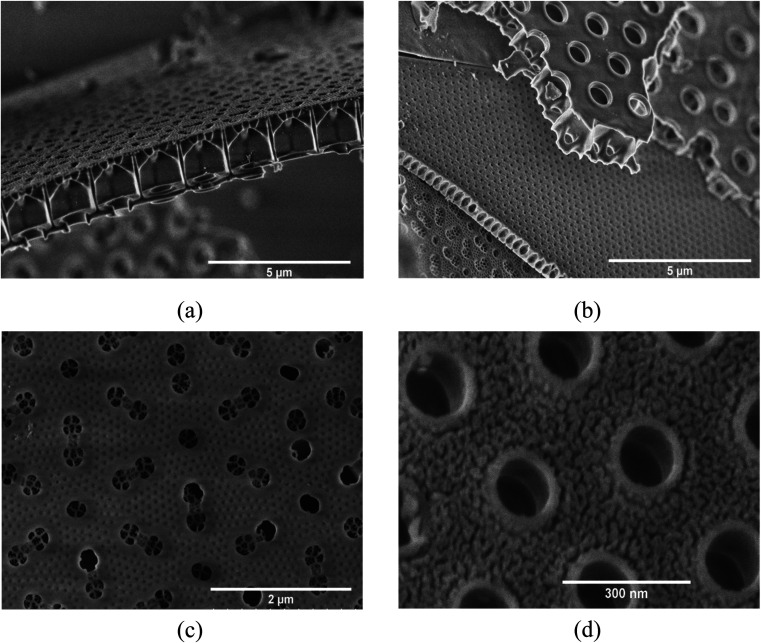
(a) Overview of multiple frustule layers, (b) cross-section of one layer of a frustule, (c) surface of frustule in (b), (d) high-resolution micrograph of frustule surface.

The microstructure and morphology of the extracted diatoms can be seen in Fig. 2(a) and (b). A magnified view of the surface in Fig. 2(b) is provided in Fig. 2(c). Fig. 2(d) shows a high-resolution micrograph of the porous surface of a frustule layer. A comparison to AFM micrographs obtained by Losic *et al.*,^34^ indicates that a large fraction of the structures is of the species coscinodiscus. The main structures of the frustules appear to be in the range of 50 nm to 3 μm, which is in agreement with Losic *et al.*^34^ In the high-resolution micrograph in Fig. 2(d), even smaller structures with dimensions below 50 nm can be seen. The frustules exhibits features ranging from roughly 3 μm to 40 nm, where the larger features originates from the frustule macro structure (different layers that make up the frustule) seen in Fig. 2(a)–(c), while the smaller features seen in Fig. 2(d) could be the fundamental building blocks of silica, as described by Schmid *et al.*^35^ In contrast to the frustule macrostructure, the smaller features might provide an interconnected mesoporous structure that goes through the entire layer.

**Fig. 3:**
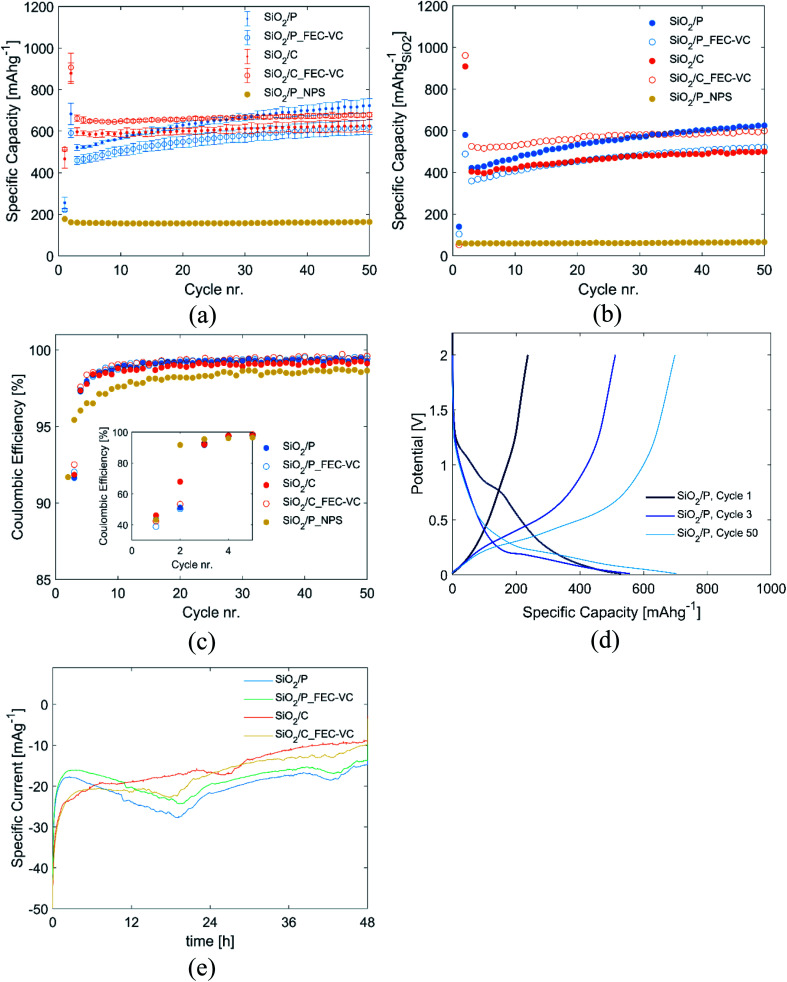
(a) Specific discharge capacity obtained for pristine (SiO_2_/P) and carbon coated (SiO_2_/C) diatomic frustules cycled in 1 M LiPF_6_ EC : DEC electrolyte or 1 M LiPF_6_ EC : DEC with 5/1 wt% FEC/VC (FEC-VC) after a 48 h potentiostatic hold step at 2 mV *vs.* Li/Li^+^ in the second cycle (with the exception of SiO_2_/P_NPS, which was not subjected to any potentiostatic hold step), (b) capacity of cells in (a) with the capacity contribution of carbon is subtracted, (c) coulombic efficiency of same cells with the first 5 cycles as insert, (d) potential profile of cycle 1, 3, and 50 for SiO_2_/P *vs.* Li/Li^+^, (e) measured current through the respective cells during the potentiostatic step.

Fig. 3(a) compares the cycling performance of electrodes fabricated from pristine milled diatom frustules (SiO_2_/P) and milled carbon coated diatom frustules (SiO_2_/C), cycled in 1 M LiPF_6_ EC : DEC electrolyte with and without additions of 5 and 1 wt% of FEC and VC and subjected to a potentiostatic step (2 mV *vs.* Li/Li^+^ for 48 h). The potentiostatic step was introduced in order to activate the SiO_2_ as previously suggest by Lepoivre *et al.*^31,35^

The potentiostatic step in the second cycle improved the capacity of all cells significantly. Initially, cells with pristine SiO_2_ (SiO_2_/P and SiO_2_/P_FEC-VC) exhibit lower capacity than cells with carbon coated SiO_2_ (SiO_2_/C and SiO_2_/C_FEC-VC). However, the cells with pristine SiO_2_ experience a greater increase in capacity over the subsequent 50 cycles. After 50 cycles, the cell with pristine SiO_2_ cycled without the FEC and VC electrolyte additives was the best performing, with a capacity of 723 mA h g^−1^. In contrast, FEC and VC appears to improve the capacity of the carbon coated SiO_2_. The capacities demonstrated here are higher than previously shown with silica derived from seawater diatom algae, where stable cycling at 523 mA h g^−1^ was demonstrated over 20 cycles (30 wt% carbon black in the electrodes), and 500 mA h g^−1^ after 80 cycles.^38–40^

For a fair comparison of the electrodes, in Fig. 3(b), the total contribution of the carbon coating (pyrolyzed sucrose) and carbon black was subtracted from the capacity and specific capacity was normalized to the mass of SiO_2_ in the electrodes. The capacity of carbon black as well as pyrolyzed sucrose was determined in separate experiments with electrodes made from the respective materials, showing stable cycling for 100 cycles at a capacity of 274 mA h g^−1^ and 560 mA h g^−1^, respectively (Fig. S2†). Comparing Fig. 3(a) and (b), the significant capacity contribution from carbon black and the amorphous carbon coating is evident, emphasizing the importance of accounting for the capacity contribution of carbon when dealing with amorphous carbon coatings. With the exception of an overall reduction in capacity, the same trends can be observed in Fig. 3(b) as in Fig. 3(a). Initially, electrodes made from the milled and carbon coated frustules cycled with FEC and VC containing electrolyte exhibits the highest capacity, which is rather stable upon cycling (maintaining a capacity of 600 mA h g^−1^ after 50 cycles). Pristine milled frustules cycled in an additive free electrolyte have lower initial capacity. However, these electrodes show improved capacity upon further cycling, increasing to 625 mA h g^−1^ after 50 cycles. The materials show remarkably good stability over the first 50 cycles with all cells exhibiting a final capacity higher than the initial. This stability is believed to originate from the formation of Li_2_O, Li_4_SiO_4_, and Li_2_Si_2_O_5_, which have been suggested to provide protection against the destructive volume expansion during lithiation of silicon.^14,25,26^

Fig. 3(c) show the corresponding coulombic efficiencies (CE) of the cells over 50 cycles. In the first cycle, CE is low for all cells, ranging from 38.9% for pristine SiO_2_ cycled in an electrolyte with FEC and VC (SiO_2_/P_FEC-VC), to 46.1% for carbon coated SiO_2_ cycled in an additive-free electrolyte (SiO_2_/C). The initially low CE may be attributed to the formation of SEI and the irreversible conversion of SiO_2_ to Si, Li_4_SiO_4_, Li_2_Si_2_O_5_ and Li_2_O. In the second cycle, the CE is still low, ranging from 50.3% to 67.9%, most likely due to additional conversion of SiO_2_ in the potentiostatic step. In the subsequent cycling, the CE increases rapidly, stabilizing around 99% after 10 cycles. It should also be noted that the cells subjected to a potentiostatic step have a significantly higher CE after 10 cycles compared to the reference cell not subjected to any potentiostatic step (SiO_2_/P_NPS). A possible explanation is that the potentiostatic step ensures that most of the SiO_2_ available for conversion indeed is converted in the first cycles, while the conversion process continues incrementally over tenths of cycles in SiO_2_/P_NPS. If SiO_2_ anodes are to be used in full cells, this is a crucial observation, as continuous depletion of Li^+^ in a full cell will rapidly deplete the reservoir of Li^+^, rendering the cell useless. In contrast, the potentiostatic step allows for the possibility of developing a pre-lithiation process, which can fully convert SiO_2_ prior to full cell assembly.

The evolution of the potential profile over cycling for SiO_2_/P can be seen in Fig. 3(d). The figure clearly displays how the potentiostatic hold step in the second cycle, results in an emerging plateau at approximately 0.2 V in the third cycle. Corresponding figures for the other cells can be found Fig. S3.†Fig. 3(e) displays the current going through the cells during the aforementioned potentiostatic step, and the integral of these curves represents the charge passed. As seen from Fig. 3(e), although the initial currents are higher for the carbon-coated samples, a higher total charge is passed during the activation step for the uncoated electrodes, and slightly higher for the conventional electrolyte compared to that with FEC and VC. The increased charge going through the cells with pristine SiO_2_ is also reflected in Fig. 3(c), showing lower CE in the 2nd cycle for the cells with pristine SiO_2_ compared to the carbon coated SiO_2_.

**Fig. 4:**
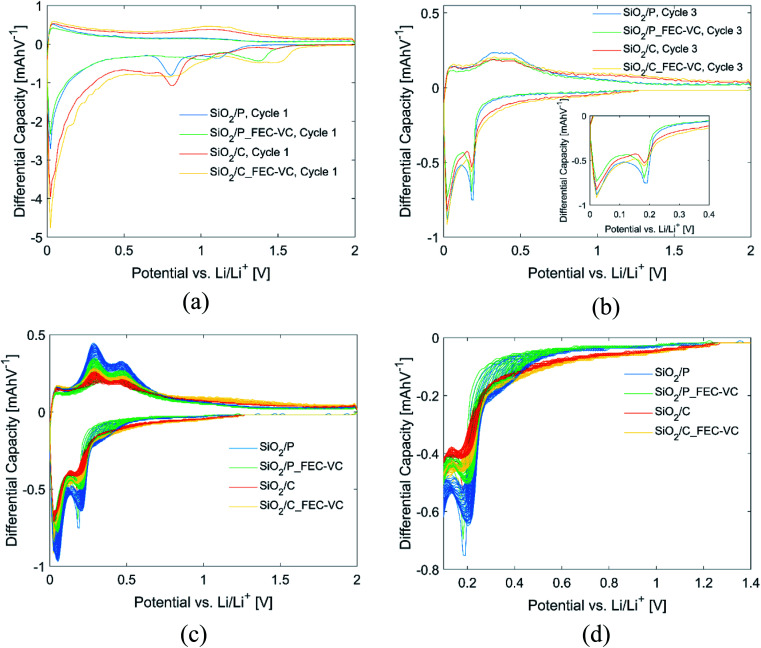
Differential capacity plots of all cells in (a) cycle 1, (b) cycle 3, (c) cycle 3–50 and (d) close-up of first cathodic peak in (c).

Further insight into the effect of carbon coating, electrolyte additives and the potentiostatic step can be gained from the differential capacity plots of the cells in Fig. 4. All cells in this figure have the same mass. In the first discharge cycle (Fig. 4(a)), electrolyte reduction peaks can be seen for all cells. In agreement with the literature, cells with FEC and VC have electrolyte reduction peaks at higher potentials, ranging between 1.4 and 1.5 V *vs.* Li/Li^+^, which appears to limit the SEI formation at 0.8 V *vs.* Li/Li^+^ associated with the reduction of EC and DEC,^15,44,45^ as may be inferred by the reduced size of these peaks. By comparing the peaks in the anodic and cathodic direction of the first cycle (Fig. 4(a)) and third cycle (Fig. 4(b)), formation of new peaks can be observed after the potentiostatic step in cycle 2. In the subsequent cycles (Fig. 4(c)), these peaks continue to develop, resulting in peaks at approximately 0.29 and 0.48 V *vs.* Li/Li^+^ in the anodic direction and 0.20 and 0.03 *vs.* Li/Li^+^ in the cathodic direction. A close-up of the first peak in the cathodic direction can be seen in Fig. 4(d). These peaks correspond quite well to the known lithiation/delithiation potentials of Si in the literature.^46^ For all cells, the peak intensities in the differential capacity plot increase over the following cycles, with the highest increase observed for the pristine sample in the conventional electrolyte, in line with the observed increase in capacity (Fig. 3(a)). Moreover, the peaks are found at the same position for each of these cycles, which is also in line with the high reversibility observed during cycling and the high CE observed after the first cycles.

**Fig. 5:**
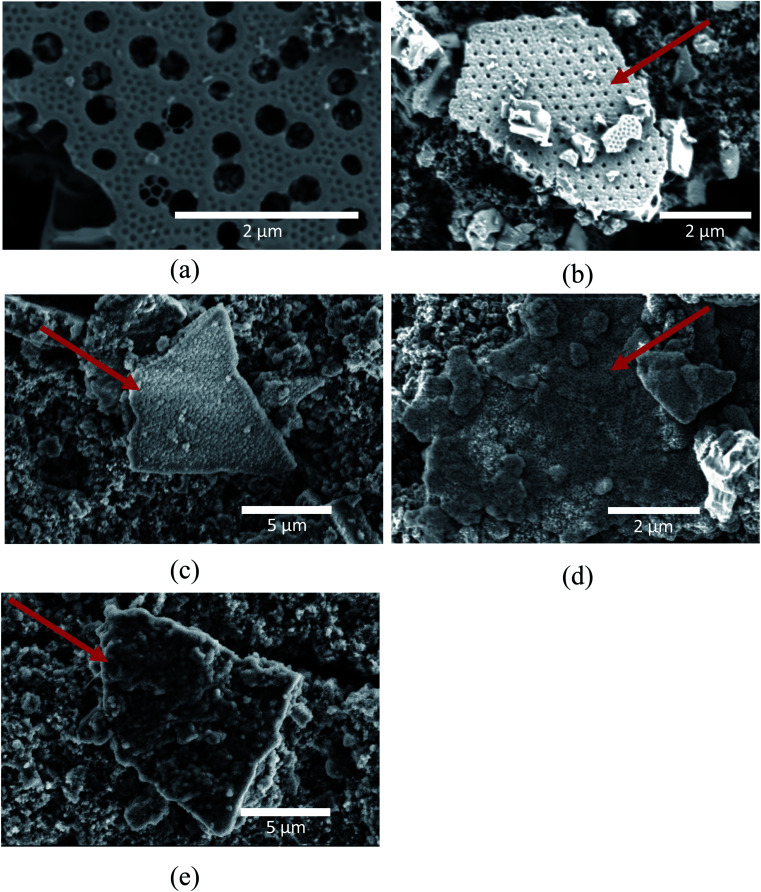
SEM micrographs of SiO_2_ particles on; (a) and (b) SiO_2_/P, (c) SiO_2_/P_FEC-VC, (d) SiO_2_/C and (e) SiO_2_/C_FEC-VC, after 50 cycles. Arrows in red indicate SiO_2_ particles.

**Fig. 6:**
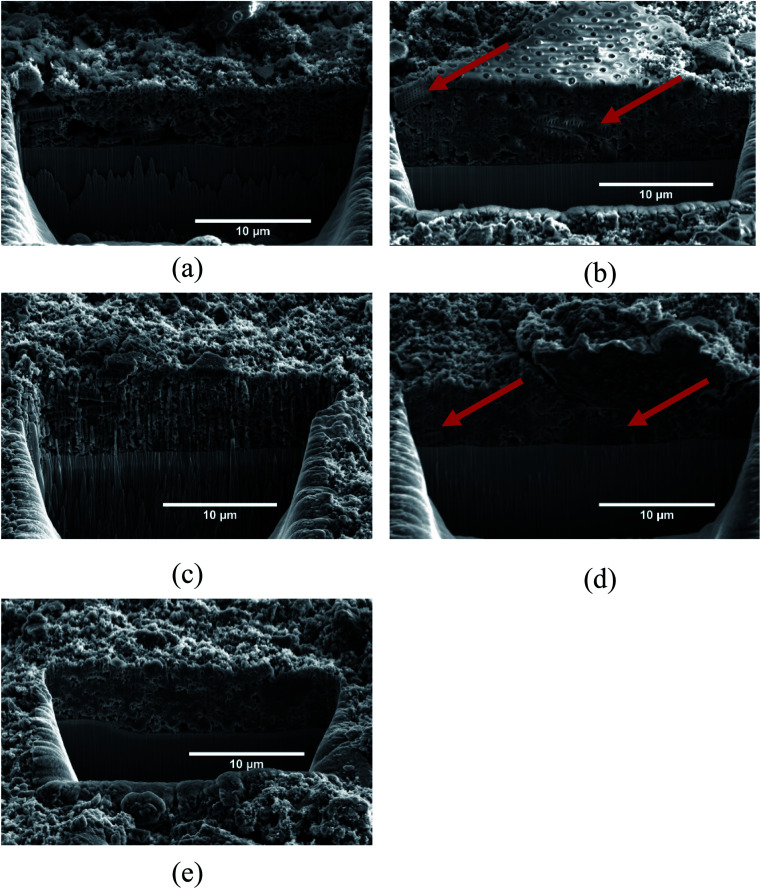
SEM micrographs of FIB cross-sections; (a) uncycled SiO_2_, (b) SiO_2_/P, (c) SiO_2_/P_FEC-VC, (d) SiO_2_/C and (e) SiO_2_/C_FEC-VC. Arrows in red indicate SiO_2_ particles.

Fig. 5 shows the SEM micrographs of the electrode surfaces after 50 cycles, and Fig. 6 shows the FIB cross-section of the same electrodes. The micrographs clearly show the deposits resulting from the SEI formation on the samples. However, the amount and morphology of the SEI appears to be dependent on both the use of carbon coating as well as the FEC and VC additives. At this point it should also be emphasized that uncycled carbon coated frustules were analyzed in the SEM, and the carbon coating was not visually evident. This suggests that all the deposits observed in Fig. 5 and 6 are SEI formation and not the carbon coating. Fig. 5(a)–(c) show electrodes with pristine SiO_2_. In this case (Fig. 5(a)), some of the SiO_2_ particles show now sign of SEI formation. Interestingly, the images of the SEI-free SiO_2_ indicates that the microstructure of the frustules is intact after cycling. To investigate the microstructure of SiO_2_ particles with substantial SEI formation, the electron beam was used to remove the layer. Fig. S4† demonstrate that the microstructure indeed is intact under the SEI layer. By comparing the indicated SiO_2_ particles in Fig. 5(b) and (c), a slight difference in the SEI morphology, depending on the electrolyte, is observed. The SEI on the electrode cycled with the electrolyte additives included (Fig. 5(c)) appears to have a rougher surface consisting of spherical particles, while the electrode cycled in the additive-free electrolyte (Fig. 5(b)) has a more uniform surface. In the case of electrodes made with carbon coated SiO_2_, the same difference in SEI morphology can be observed in cells with (Fig. 5(e)) and without (Fig. 5(c)) the additives. However, in both cases, the carbon coating appears to increase the thickness of the SEI layer significantly. The increased SEI formation on carbon coated SiO_2_ can also be observed within the electrode in the FIB cross-section micrographs in Fig. 6. Comparing the electrodes with pristine SiO_2_ in Fig. 6(b) with the electrodes with carbon coated SiO_2_ in Fig. 6(d), the structure of the pristine SiO_2_ particles can be easily observed, while the increased SEI formation makes it difficult to spot individual carbon coated SiO_2_ particle. Moreover, the overall porosity of the electrode appears to be better preserved in the electrodes cycled in the electrolyte containing additives (Fig. 6(c) and (d)), compared to the additive-free electrolyte (Fig. 6(b) and (e)). The observation that the CE for pristine SiO_2_ is lower than that of the carbon coated SiO_2_, and that less deposition products can be observed on the pristine SiO_2,_ suggest that the conversion of SiO_2_ could be higher in electrodes with pristine SiO_2_. This hypothesis is further strengthened by the data from galvanostatic cycling in Fig. 3(a), where SiO_2_/P exhibits the highest capacity after 50 cycles. Moreover, the SEM images might provide insight into the effect of FEC and VC as electrolyte additives. In cells with pristine SiO_2_, FEC and VC appears to limit the performance of the cells, while the opposite is true for carbon coated SiO_2_. One possible hypothesis is that the initial decomposition of FEC and VC observed in Fig. 4(a) increases the thickness and/or density of the SEI formed on the pristine SiO_2_, which again might hamper the conversion of SiO_2_. On the other hand, carbon coated SiO_2_ shows quite extensive SEI formation in Fig. 5(d) and (e). In this case, the initial decomposition of FEC and VC might limit the overall SEI formation. This hypothesis is supported by the differential capacity plots in Fig. 4(a), which clearly show that the initial reduction peak of FEC at approximately ∼1.45 V *vs.* Li/Li^+^ suppress the reduction peak of EC/DEC at 0.8 V *vs.* Li/Li^+^.

The good electrochemical performance of the diatom frustules is most likely related to the specific porous structure of the diatom frustules, expected to be beneficial to the anode performance. The pores might ease the electrolyte penetration into the electrode and improve the accessibility of the electrolyte and the reacting Li^+^ ions towards the active material. In addition, the structure might also buffer any expansion of lithiated Si during cycling and thereby improve the cycling stability. This is actually observed in Fig. 5, where the SEM micrographs show very little distortion of the superstructure for the cycled electrodes. Similar findings have also been reported in the literature, for instance for amorphous SiO_2_/C composite anodes.^21^ In this work, the good electrochemical performance was attributed to the porous structural characteristics of the SiO_2_/C material, allowing more electrolyte to be absorbed into active materials, and also contributing to good rate-capability and cycling stability of the electrode. Similar conclusions were made in ref. 36, where the low charge transfer resistance of C@Si–SiO_2_ electrodes was attributed to the mesoporous structure, resulting in good electrolyte–electrode contact.

**Fig. 7:**
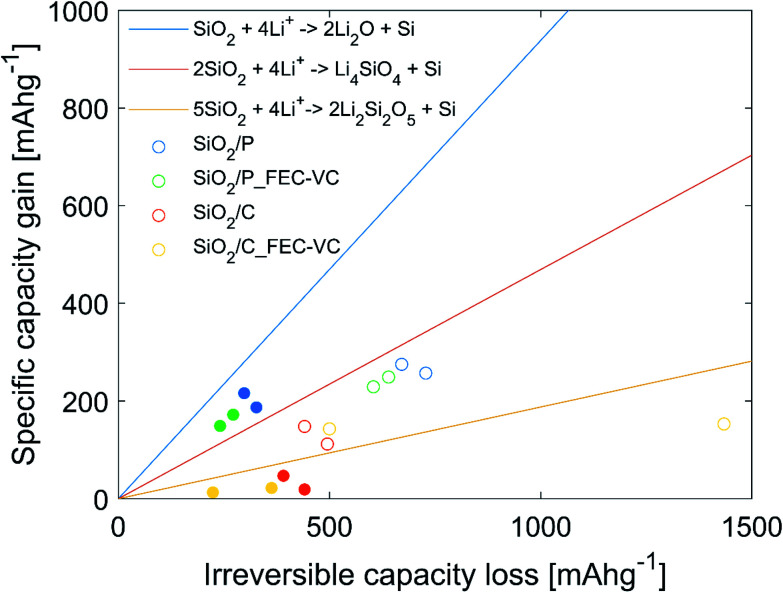
Increase in specific capacity (*Q*_G_) as a function of ICL. The three lines (yellow, red and blue) show the theoretical capacity gain as a function of ICL for the three proposed lithiation mechanisms of SiO_2_. The scatter dots show the measured increase in capacity as a function of the ICL over the electrochemical reduction step, cycle 2 to cycle 3 (hollow), and over cycle 3 to cycle 50 (filled).

In an effort to identify which of the three proposed conversion reactions of SiO_2_ is taking place, the specific capacity gain (*Q*_G_), given by the increase in capacity for a set of cycles, was plotted against the irreversible capacity loss (ICL), given by the sum of the CE losses for the same set of cycles. This method is based on the work of Lepoivre *et al.* and the data is plotted in Fig. 7.^31^ In addition, Fig. 7 includes the theoretical capacity gain *vs.* irreversible capacity loss calculated for the conversion reactions (1) to (3), representing the maximum possible increase in specific capacity possible for a certain ICL. These theoretical values are found by calculating the theoretical specific capacity for reaction (1) to (3) (equaling 335, 836 and 1673 mA h g^−1^, respectively) and the corresponding theoretical ICL for total conversion (equaling 1784 mA h g^−1^ for all the reactions). It should be noted that experimental results will always include some contribution from irreversible losses due to side reactions, thus the experimental *Q*_G_ values will always be lower than the theoretical limit for a given ICL. The experimental values plotted as dots in Fig. 7, represent the increase in capacity from cycle 2 to cycle 3 (hollow), as well as over cycle 3 to cycle 50 (filled), plotted against the ICL for the same cycles. Cycle 1 was excluded, as a large fraction of the ICL loss in this cycle may be assumed to be caused by SEI formation. In Fig. 7, all the points representing *Q*_G_ for the cycle 2 to 3 are below the theoretical line of conversion reaction (2), but above the line of conversion reaction (3). It is therefore reasonable to believe that the capacity gain during the potentiostatic step is mainly driven by reaction (2). From cycle 3–50, *Q*_G_ for the cells with pristine SiO_2_ is well above the theoretical limit of reaction (2), implying a significant contribution from reaction (3). In the case of cells with carbon coated SiO_2_, virtually no capacity increase is seen in cycle 3–50. Thus, it is likely that most of the ICL is caused by SEI formation. SEM micrographs of the electrode surface after cycling (Fig. 5(d) and (e)) showing decompositions products on the surface, also support this.

In previous work on SiO_2_ frustules as an anode material, where stable cycling (521 mA h g^−1^ for 20 cycles) was demonstrated with carbonized diatom algae, Li_2_O could not be detected on the surface after cycling.^38^ Thus, indicating that the conversion most probably occurs *via* a lithium silicate, implying a lower theoretical capacity.

## 4. Conclusions

In this work, it is demonstrated that diatom frustules from the seabed, subject only to a simple cleansing and milling procedure, exhibit good performance as anodes for Li-ion batteries. The best performance was obtained after an initial activation procedure, implying a potential hold step at 2 mV for 48 h. The highest capacity (723 mA h g^−1^) was obtained with composite electrodes with diatom frustules and conventional carbon black conductive additive, with the capacity still increasing after 50 cycles. The capacity was around 624 mA h g^−1^ after subtraction of the contributions from the carbon black. Carbon coated diatom frustules showed a slightly lower and stable capacity after 50 cycles (679 mA h g^−1^, 600 mA h g^−1^ after subtraction of contributions from the carbon coating and the carbon black). Addition of FEC to the electrolyte improved the performance of the carbon coated frustules, but not the pristine material. The good performance was attributed to an efficient activation of the material upon cycling, with a gain in capacity compatible with a high fraction of material converted to Si and Li_2_O. After the initial formation cycles a high coulombic efficiency was observed, corresponding to low formation of electrolyte decomposition products as compared to the carbon coated material.

## Conflicts of interest

We declared that there is no conflict of interests and all the listed authors fully agree with publishing the manuscript.

## Supplementary Material

RA-009-C9RA07271C-s001
