# Supplementary material for: Silica from diatom frustules as anode material for Li-ion batteries
Source: RSC Adv. 2019 Dec 12;9(70):41228–39. doi: 10.1039/c9ra07271c (PMC9076370; doi:10.1039/c9ra07271c)
Supplement: RA-009-C9RA07271C-s001 [file RA-009-C9RA07271C-s001.pdf]

Electronic Supplementary Material (ESI) for RSC Advances.
This journal is © The Royal Society of Chemistry 2019

Supplementary information for

Silica from diatom frustules as anode material for Li-ion batteries

Andreas Nicolai Norberg [a], Nils Peter Wagner [a,b], Henning Kaland [a],
Fride Vullum-Bruer [c], Ann Mari Svensson [a].

Silica from diatom frustules as anode material for Li-ion batteries

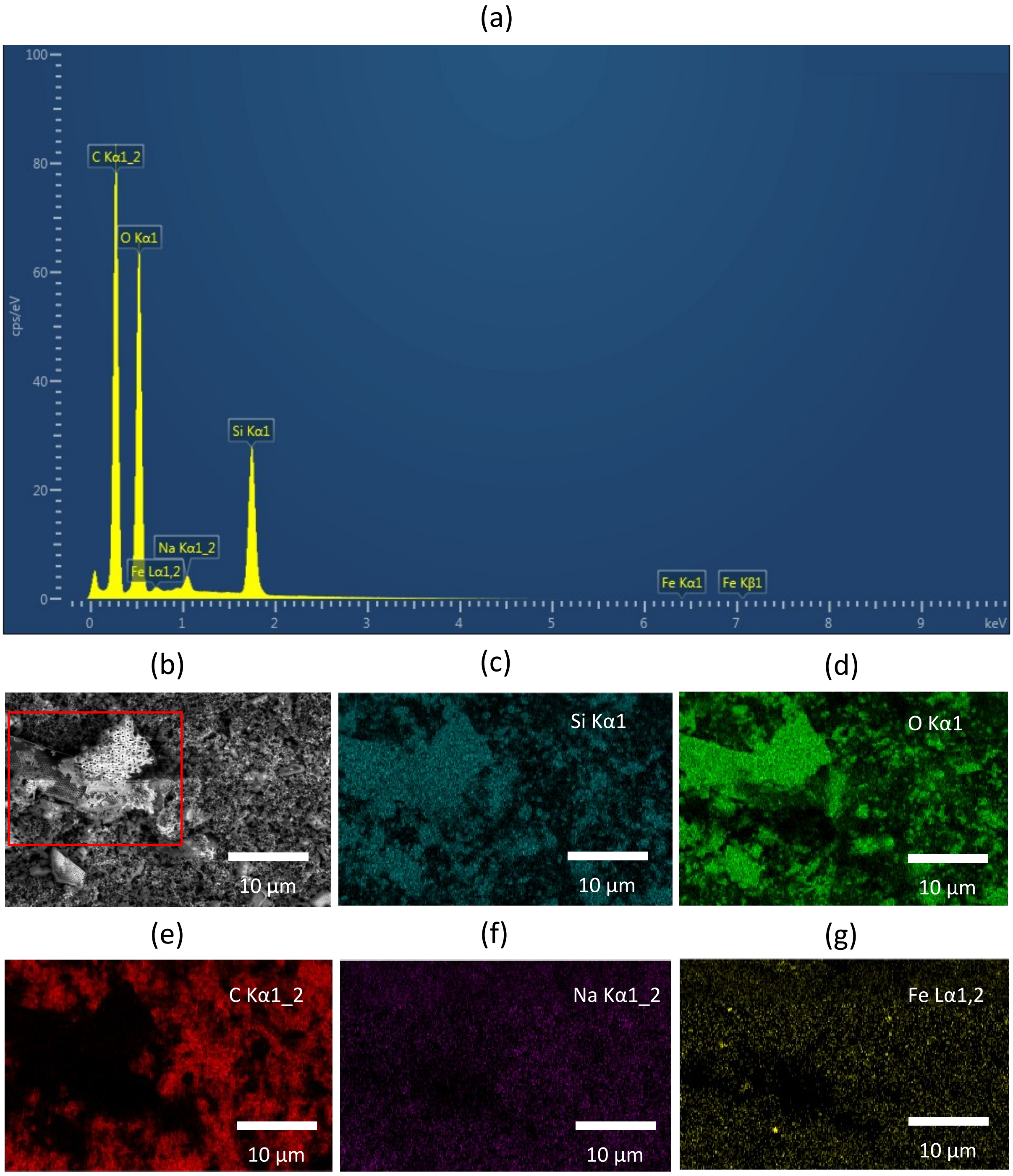

Fig. S1: EDX of uncycled SiO_2/P electrode. (a) Full spectrum, (b) SEM image of area analyzed with largest visible SiO_2 particle demarked, (c) Si Kα1 signal, (d) O Kα1 signal, (e) C Kα1_2 signal, (f) Na Kα1_2 signal, (g) Fe Lα1,2 signal.

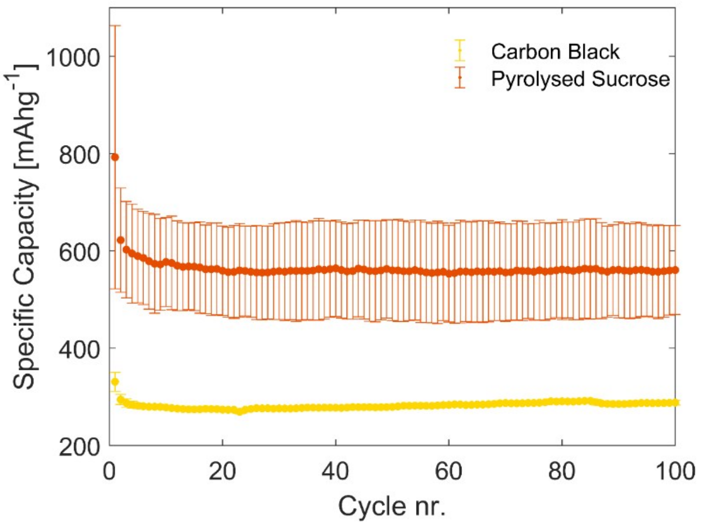

Fig. S2: Capacity of carbon reference cells.

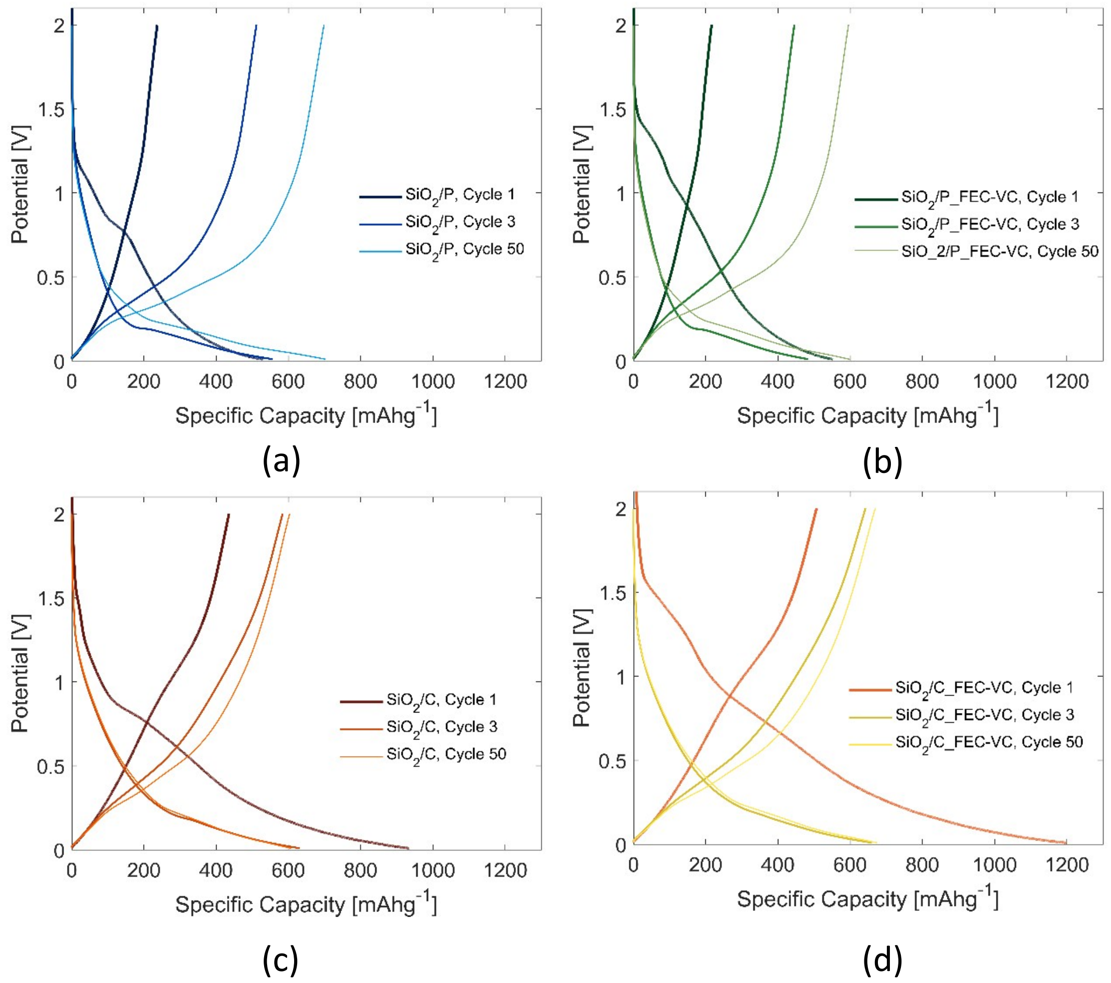

Fig. S3: Potential profile of cycle 1, 3 and 50 for (a) SiO2/P, (b) SiO2/P_FECVC, (c) SiO2/C, (d) SiO2/C_FECVC.

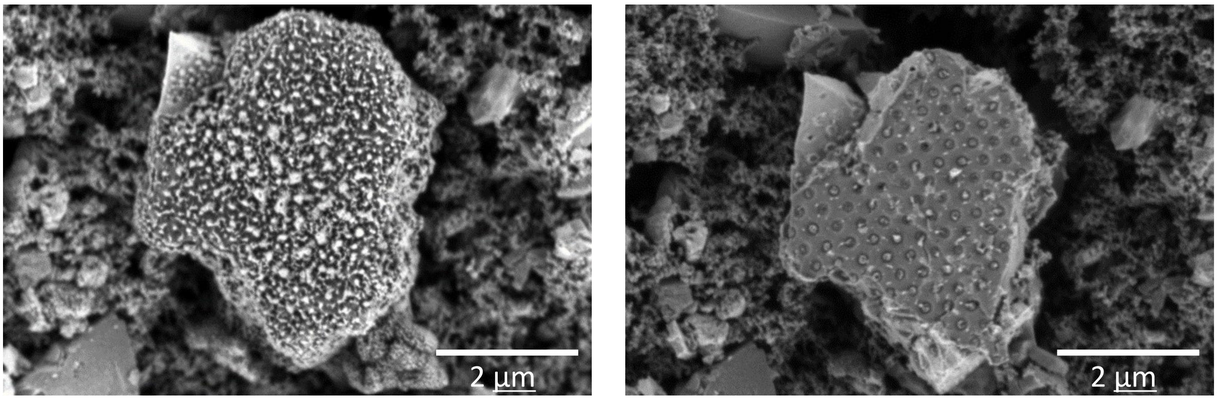

(a) (b)

Fig. S4: SEM images of SiO2 particle (a) before and (b) after removal of SEI with electron beam.
